# The Effect of Neoadjuvant Chemotherapy Combined With Brachytherapy Before Radical Hysterectomy on Stage IB2 and IIA Cervical Cancer: A Retrospective Analysis

**DOI:** 10.3389/fonc.2021.618612

**Published:** 2021-03-23

**Authors:** Yun Dang, Qing Liu, Lixia Long, Hua Luan, Qingfang Shi, Xunyuan Tuo, Shumei Tuo, Yilin Li

**Affiliations:** Gansu Provincial Maternity and Child Care Hospital, Lanzhou, China

**Keywords:** local advanced, cervical cancer, neoadjuvant chemotherapy, operation, brachytherapy

## Abstract

**Objective:**

This study aims to retrospectively evaluate and compare the clinical efficacy in patients with stage IB2 and IIA cervical cancer, who treated with neoadjuvant chemotherapy combined with brachytherapy or not before radical hysterectomy.

**Methods:**

The data of patients who have diagnosed with stage IB2 and IIA cervical cancer between January 2010 and December 2013 were retrieved through the Hospital Information System (HIS) of Gansu Provincial Maternal and Child Health Hospital. Patients were divided into two groups: neoadjuvant chemotherapy combined with brachytherapy followed by radical hysterectomy group (NACT+BT Group) and direct radical hysterectomy group (RH Group). The rate of adjuvant radiotherapy, progression-free survival (PFS), and overall survival (OS) were compared between the two groups.

**Results:**

A total of 183 patients were included in this study with 82 in the NACT+BT group and 101 in the RH group. The median follow up duration was 44.9 months for the NACT+BT group and 38.1 months for the RH group. The 5-year PFS for NACT+BT Group was 93.8%, which was significantly higher compared to the RH group (77.2%, *P*= 0.0202). The rate of postoperative adjuvant pelvic radiotherapy was significantly lower in the NACT+BT group compared to the RH group (30.49% vs 79.21%; *P <*0.05). COX multivariate analysis showed that NACT+BT increased PFS by 29% compared with RH treatment, and Positive margin decreased PFS and OS by by 4.7 and 6.87 times, respectively.

**Conclusion:**

Neoadjuvant chemotherapy combined with brachytherapy followed by radical hysterectomy (NACT+BT) can extend PFS, reduce postoperative pathological risk, and postoperative adjuvant pelvic radiotherapy compared to the direct radical hysterectomy (RH).

## Introduction

Cervical cancer is the second most common cancer and the third leading cause of cancer-related deaths among women in less developed countries. Worldwide, there are 5.27 million new cases and 2.65 million deathseach year (1), and nearly 90% of cervical cancer deaths are recorded in developing countries due to the inadequate public health service system and limited coverage of cervical cancer screening (2). The International Federation of Gynecology and Obstetrics (FIGO) defines the tumor categorized in stage Ib2 through stage III as locally advanced cervical cancer (LACC) in 2009 (3). LACC is characterized by an increased lymph node metastasis, uterine tumor invasion, and vasculature tumor, whereas the 5-year survival rate is around 50% to 70% (4).

Although the National Comprehensive Cancer Network (NCCN) guidelines recommend chemoradiation for LACC, therapeutic methods vary greatly in different parts of the world. In North America, chemoradiation is the most popular method, whereas in Europe, Asia, and Latin America, neoadjuvant chemotherapy combined with brachytherapy followed by radical hysterectomy is the main therapeutic approach. Radical hysterectomy can reduce the damage of ovarian function, maintain maximum vaginal length and elasticity, and improve patient’s immediate and long term survival quality. In addition to keeping the postoperative pathological primitive state, which is conducive to comprehensive and accurate guidance of postoperative adjuvant therapy after surgery. According to the cervical cancer FIGO guidelines, In the present study, we explored whether neoadjuvant chemotherapy combined with brachytherapy can 1) reduce the rate of supplementary pelvic radiotherapy and chemotherapy and 2) improve the survival in patients with stage IB2 and IIA cervical cancer (5).

## Materials and Methods

### Study Population

This study was approved by the ethical committee in Gansu Provincial Maternity and Child Care Hospital, China. The data of 183 patients who were diagnosed with stage IB2 and IIA cervical cancer between January 2010 and December 2013 were retrieved through the HIS system of Gansu Provincial Maternal and Child Health Hospital. These patients were included in this study because they all met the following criteria: (1) patients with squamous cell carcinoma; (2) clinical stage Ib2 and IIa defined by the FIGO 2009; (3) age between 18 and 75 (years); (4) American Eastern Cooperative Oncology Group (ECOG) score 0–2 points; (5) with normal liver and kidney function: serum transoxidase (AST, ALT) lower than 40 lU/ml, total bilirubin <5 mg/dL, and urea nitrogen <20 mg/dL; 6) normal bone marrow function: neutrophil count >1,500/mm^3^ and platelet >100,000/mm^3^. At the same time, none of these patients meet the following exclusion criteria: (1) patients with adenocarcinoma and adenoid carcinoma; (2) patients with a history of radiotherapy or chemotherapy; (3) history of cancer; (4) pregnancy; (5) dysphonia. In the Neoadjuvant chemotherapy combined with brachytherapy followed by radical hysterectomy group (NACT+BT group), patients must complete both NACT+BT and radical hysterectomy. If the disease progresses during NACT+BT without completing radical hysterectomy, it will not be included in NACT+BT Group.

### Clinical Data

All the clinical data were obtained through the Hospital Information System (HIS). The historical treatment data of 183 patients were analyzed and divided into two groups according to whether neoadjuvant chemotherapy was used before radical hysterectomy: neoadjuvant chemotherapy combined with brachytherapy group (NACT+BT group), direct radical hysterectomy (RH group).

Patients in the RH group received Piver type III radical hysterectomy and pelvic lymph node excision. Patients in the NACT+BT group received cisplatin 75 mg/m^2^ combined with paclitaxel 135 to 175 mg/m2 every 3 weeks during routine chemotherapy (patients were treated with 192Ir of after loading vaginal brachytherapy, the dose was 15 Gy at point A that performed in three times within 3 weeks) followed by completing Piver type III radical hysterectomy. The postoperative pathological factors included positive margin, lymph node metastasis, parametrial extension, deep stromal invasion, lymphovascular space invasion, and large tumor diameter.

### Assessment and Follow-Up

After the patient has received treatment, imaging examinations (CT or PET-CT, etc.) will be performed every 3 or 6 months. Clinical response was based on the Response Evaluation Criteria in Solid Tumor (RESICT v1.1). Complete remission (CR) was established if no tumor was observed. Partial response (PR) was defined if the maximal diameter of the lesion was reduced by more than 30%. Progressive disease (PD) was defined if the maximum diameter of the lesion was increased by more than 20% or new lesions were detected. Patients who did not achieve PR or PD were assessed as stable disease (SD) and defined as non-responders, whereas patients with CR and PR were defined as adjuvant therapy responders (6).

Follow-up data were obtained through outpatient medical records, and by consulting the doctor and the patient’s family members. Overall surviva(l (OS) was measured from the date of registration to the date of death from any cause, and data were censored at the time of the last follow-up for surviving patients. Progression-free survival (PFS) was measured from the date of randomisation to the date of the first event, and data were censored at the last date on which the absence of disease progression was confirmed.

### Statistical Analysis

SAS 9.4 software was used for statistical analysis. The countable data were analyzed using the chi-square test or Fisher’s exact probability method; the quantitative data were represented by x ± s and were analyzed usingt -test. The 5-year PFS was analyzed using the Kaplan-Meier method, and the log-rank test was used for evaluation of the group differences. P<0.05 was considered statistically significant.

## Result

### General Information

We present the basic demographic and clinical factors in patients with LACC. No statistical difference was observed regarding the age at diagnosis, BMI, stage (Ib2, IIa), preoperative complications (diabetes), and preoperative tumor diameter between the two groups (*P*<0.05). Higher bleeding volume was found in the NACT+BT group compared to the RH group. In addition, surgical complications (urinary system injury, deep venous thrombosis) were different between the two groups (**Table 1**).

**Table 1:** Basic demographic and clinical factors in patients with local advanced cervical cancer (LACC).

	NACT+BT Group (n = 82)	RH Group (n = 101)	*P*
Age	48.21 ± 10.16	49.15 ± 9.23	0.5186
BMI	24.09 ± 2.12	23.18 ± 1.37	0.9998
Stages			
IB2	43 (52.44%)	41 (40.40%)	0.1098
IIA	39 (47.56%)	60 (59.41%)	
Diabetes			
Yes	10 (12.20%)	8(7.92%)	0.3358
No	71 (87.80%)	92(92.08%)	
Tumor diameter (cm)	4.89 ± 1.25	4.85 ± 1.26	0.9415
Bleeding volume (ml)	364.74 ± 245.93	214.17 ± 173.63	<0.01
Deep venous thrombosis			
Yes	7 (8.54%)	5(4.95%)	0.3312
No	75 (91.46%)	96(95.05%)	
Urinary system injury			
Yes	3 (3.66%)	4(3.96%)	0.2978
No	79 (96.34%)	97(96.04%)	

BMI = weight(kg)/height, (m^2^).

### New Adjuvant Treatment Effectiveness

In the NACT+BT group, there were 73 cases with PR and no cases with CR. In addition, the size of the tumor was significantly reduced in the NACT+BT group compared to the RH group (**Table 2**).

**Table 2:** Comparison of therapy responses in patients.

	CR	PR	SD	PD	Total	Tumor diameter (cm)	*P*
RHGroup	\	\	\	\	101	4.85 ± 1.26	
NACT+ BTGroup		73 (89.02%)	7 (8.54%)	2 (2.44%)			<0.0001
	\				82	1.32 ± 0.81	

After the patient has received treatment, tumor evaluation was performed every 3 or 6 months.

### Surgical Pathological Data

There were no statistically significant differences in the number of lymph node excision, tumor diameter, lymph node metastasis, the positive margin between the two groups (*P*>0.05); while significantly decreased deep stromal invasion, parametrial extension and lymphovascular space invasion were found in NACT+BT group compared to RH group (*P*<0.05) (**Table 3**).

**Table 3:** Comparison of surgical pathological factors.

	NACT+BT Group (n = 82)	RH Group (n = 101)	*P*
**Number of lymph nodes**	28.16 ± 4.16	28.77 ± 4.15	0.3312
**Tumor diameter (cm)**	1.27 ± 0.82	4.85 ± 1.26	<0.0001
**Lymph node metastasis**			
Yes	11(13.41%)	21(20.80%)	0.1965
No	71(86.59%)	80(79.20%)	
**Deep stromal invasion**			
Yes	35(42.68%)	69(68.32%)	0.0005
No	47(53.32%)	32(31.68%)	
**Parametrial extension**			
Yes	2(2.44%)	13(12.87%)	0.0065
No	80(97.56%)	88(87.13%)	
**Lymphovascular space invasion**			
Yes	12(14.63%)	38(37.62%)	0.0005
No	70(85.37%)	63(62.38%)	
**Positive margin**			
Yes	6(7.32%)	9(8.91%)	0.6948
No	76(92.68%)	92(91.08%)	

### Postoperative Adjuvant Radiotherapy

The ratio of postoperative adjuvant pelvic radiotherapy was 30.49% in the NACT+BT group and 79.21% in the RH group, and the difference was statistically significant (*P* <0.0001) (**Table 4**).

**Table 4:** Postoperative supplementary radiotherapy.

NACT+BT Group	RH Group	T	P
(n = 82)	(n=101)		
25 (30.49%)	80 (79.21%)		
		43.93	<0.0001
57 (69.51%)	21(20.79%)		

### Survival Data

**Figure 1:**
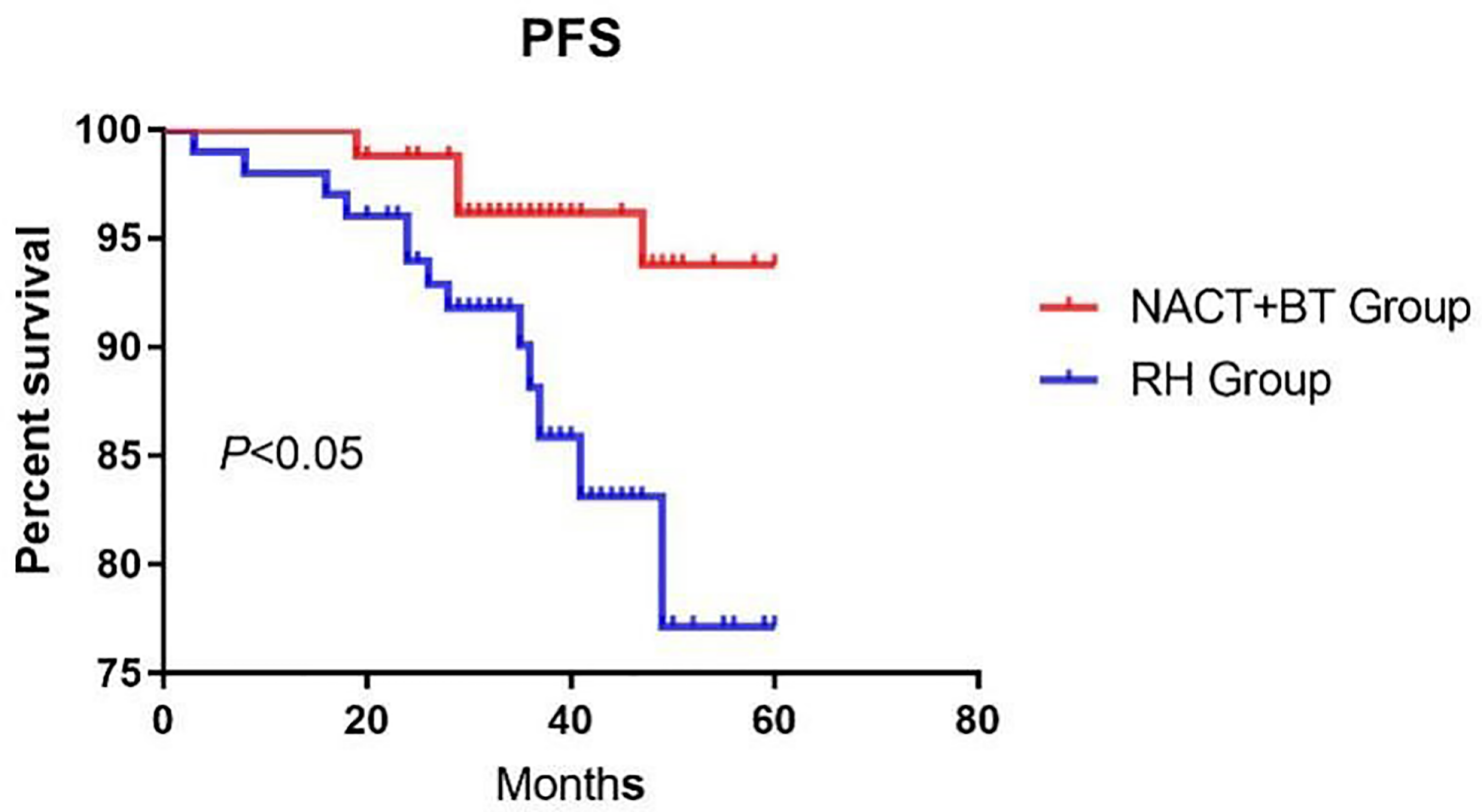
Progression-free survival.

**Figure 2:**
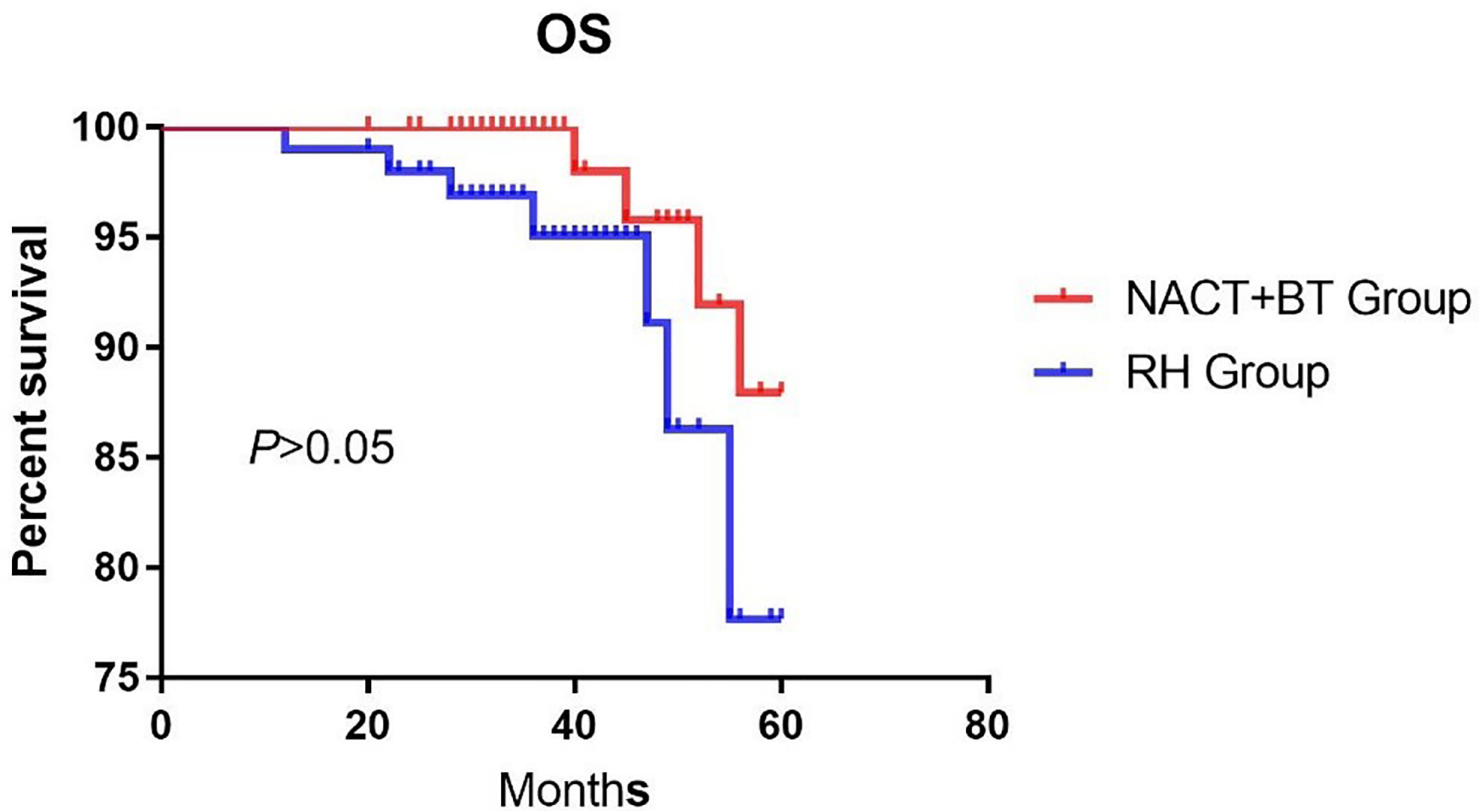
Overall survival.

The median follow-up time was 41.16 months (12–67 months). There were 17 recurrent cases in total; 4 cases in NACT+BT Group, and 13 cases in the RH group; and a total of 10 fatal cases, 3 cases in the NACT+BT group, and 7 cases in RH group. The 5-year PFS and OS in NACT+BT group vs RH group were 93.8% vs 77.2% (95% CI 0.1129–0.7565, *P*= 0.0202), 87.9% vs 77.7% (95%CI 0.1187–1.314, *P*= 0.1162) (**Figures 1** and **2**).

### COX Multivariate

COX multivariate analysis showed that NACT+BT increased PFS by 29% compared with RH treatment, and Positive margin decreased PFS and OS by by 4.7 and 6.87 times, respectively (**Table 5**).

**Table 5:** Multifactor analysis of factors affecting 5-year progression-free survival (PFS) and overall survival (OS) in local advanced cervical cancer (LACC).

	PFS						OS					
	B	SB	Wald	HR	95% CI	*P*	B	SB	Wald	HR	95% CI	*P*
Age												
<40 years	0.53	0.57	0.87	1.71	0.577–5.236	0.35	0.35	0.85	0.17	1.43	0.627–7.614	0.68
≥45years												
Therapy method												
NACT+BT	- 1.22	0.58	4.49	0.29	0.095–0.931	0.03	- 0.63	0.76	0.67	0.54	0.120–2.388	0.41
RH												
Stages												
IB2	0.18	0.49	0.13	1.20	0.457–3.133	0.72	- 0.20	0.75	0.07	0.82	0.188–23.692	0.79
IIA												
Positive margin												
Yes	1.55	0.53	8.41	4.70	1.625–13.377	0.00	1.93	0.61	9.90	6.86	2.067–22.792	0.00
No												
Lymph node metastasis												
Yes	0.72	0.55	1.68	2.05	0.69–6.05	0.19	0.32	0.73	0.20	0.66	0.322–5.746	0.66
No												
Deep stromal invasion												
Yes	0.75	0.70	1.15	2.11	0.54–8.36	0.28	1.55	1.12	1.93	4.71	0.529–41.946	0.17
No												
Lymphovascular space invasion												
Yes	0.60	0.49	1.47	1.82	0.691–4.782	0.23	-0.35	0.69	0.26	0.70	0.182–2.712	0.61
No												
**Parametrial extension**												
Yes	1.14	0.64	3.16	3.11	0.891–10.871	0.08	0.14	1.13	0.02	1.16	0.127–10.536	0.90
No												

## Discussion

According to NCCN guidelines, chemoradiation is recommended for the management of LACC. Nonetheless, in the developing countries, surgical treatment is still used to treat a large number of locally advanced cervical cancers, and regardless of the type of treatment, the five-year survival rate of patients with l LACC is around 50% to 70% (3). Preoperative neoadjuvant chemotherapy combined with brachytherapy and direct surgery have been continuously applied in clinical practice, and numerous studies have reported that neoadjuvant chemotherapy combined with brachytherapy might benefit patients with LACC regarding PFS, but the overall survival failed to reach satisfactory results. In addition, preoperative neoadjuvant therapy might affect the ability to find the palace of infiltration and the tiny lymph node metastases, leading to recurrence (7).

The advantages of radical hysterectomy are that it minimizes the damage to the ovary, maintaining maximum vaginal length and elasticity, thereby improving the patient’s immediate and long-term survival, and maintaining postoperative pathology in original state. It is also beneficial for comprehensive and accurate guidance of postoperative adjuvant therapy after surgery. Nonetheless, in the vast majority of cases, pelvic radiotherapy and chemotherapy are still necessary, and this “sequential” therapy reduces the advantages of surgery (8). Based on previously published studies, more than 60% of patients with LACC treated by direct surgical treatment require pelvic radiotherapy after the operation (9), which can eventually lead to adverse long-term consequences, such as gastrointestinal symptoms, urinary system symptoms and hematologic complications (10, 11). The incidence of 3-degree bone marrow suppression has shown to be 18.3%, and the incidence of 4-degree myelosuppression was 22% (12, 13). In the present study, around 80% of cases needed pelvic radiotherapy after direct radical hysterectomy. Huguet and Modarress have indicated that neoadjuvant chemotherapy combined with brachytherapy could be used to control local lesions compared with direct radical hysterectomy; nonetheless, the survival rate was not significantly improved (14, 15). In 2012, a Cochran evaluation system indicated that neoadjuvant chemotherapy combined with brachytherapy can reduce the rate of lymph node metastasis (HR = 0.54, P < 0.05), and parametrial extension (HR = 0.52, *P* < 0.05), and can improve PFS (HR = 0.76, *P* < 0.05), but it cannot improve OS (16). Our study showed that neoadjuvant chemotherapy combined with brachytherapy was more effective than direct radical hysterectomy regarding local lesions (1.32 ± 0.81cm vs. 4.85 ± 1.26cm, *P*<0.05); compared with direct radical hysterectomy it revealed reduced deep stromal invasion (42.68% vs. 68.32%, *P*<0.05), parametrial invasion (2.44% vs. 12.87%, *P*<0.05) and lymphovascular space invasion (14.63% vs. 37.62%, *P*<0.05). Nevertheless, neoadjuvant chemotherapy combined with brachytherapy did not affect lymph node metastasis and positive margin compared with direct radical hysterectomy. The PFS in the direct radical hysterectomy group was significantly lower compared to the neoadjuvant chemotherapy combined with brachytherapy group(*P*<0.05), COX multivariate analysis showed that NACT+BT increased PFS by 29% compared with RH treatment, but there was no significant difference in the OS between the two groups. Also, the rate of postoperative adjuvant pelvic radiotherapy was significantly lower, and it could prevent long term complications from pelvic radiation therapy and improve the quality of life of LACC patients.

In short, preoperative neoadjuvant chemotherapy combined with brachytherapy can improve pathological factors related to postoperative risk and PFS, thus having a long term beneficial effect on the patient’s quality of life. Our study needs to be confirmed in large cohort studies. In addition, the amount of minimally invasive surgery in this study was very small, so it was not included in this study scope. It is undeniable that minimally invasive surgery can play an important role (17–19), so in future studies, we will pay attention to the impact of minimally invasive surgery.

## Data Availability Statement

The raw data supporting the conclusions of this article will be made available by the authors, without undue reservation. Requests to access the data sets should be directed to 34370100@qq.com.

## Ethics Statement

This study was approved by the Institutional Ethics Committee of the Gansu Provincial Maternity and Child-care Hospital, China (2013-11).

## Author Contributions

YD: data collection, data analysis, and manuscript writing/editing. QL: protocol/project development. LL: protocol/project development. HL: protocol/project development. QS: protocol/project development. XT: data collection. ST: data collection. YL: data collection and data analysis. All authors contributed to the article and approved the submitted version.

## Funding

This study was supported by Gansu Provincial Maternity and Child-care Hospital.

## Conflict of Interest

The authors declare that the research was conducted in the absence of any commercial or financial relationships that could be construed as a potential conflict of interest.

## Abbreviations

LACC, local advanced cervical cancer; FIGO, International Federation of Gynecology and Obstetrics; NCCN, National Comprehensive Cancer Network; HIS, Hospital Information System.
